# Supplementary material for: Characterization of acquired β-lactamases in Pseudomonas aeruginosa and quantification of their contributions to resistance
Source: Microbiol Spectr. 2024 Sep 9;12(10):e00694-24. doi: 10.1128/spectrum.00694-24 (PMC11448201; doi:10.1128/spectrum.00694-24)
Supplement: Figures S1 and S2 — Figure S1: Extended phylogenetic tree. Figure S2: Maps of acquired genetic elements. [file spectrum.00694-24-s0001.pdf]

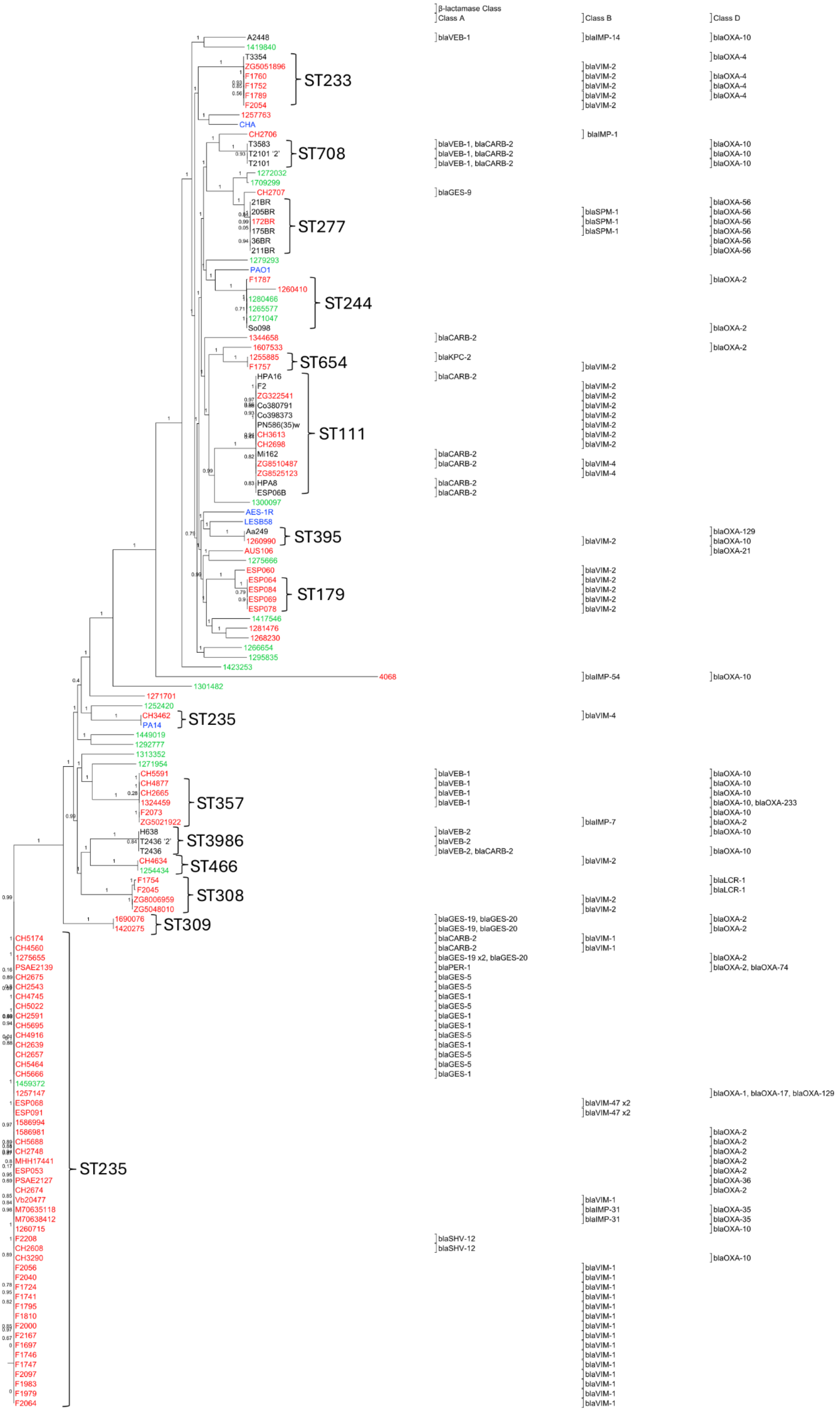
β-lactamase Class
Class A
Class B
Class D
A2448
blaVEB-1
blaIMP-14
blaOXA-10
1419840
blaOXA-4
T3354
ZG5051896
blaVIM-2
F1760
blaVIM-2
blaOXA-4
F1752
blaVIM-2
blaOXA-4
F1789
blaVIM-2
blaOXA-4
F2054
blaVIM-2
ST233
1257763
CHA
CH2706
blaIMP-1
T3583
blaVEB-1, blaCARB-2
blaOXA-10
T2101 '2'
blaVEB-1, blaCARB-2
blaOXA-10
T2101
blaVEB-1, blaCARB-2
blaOXA-10
ST708
1272032
1709299
CH2707
blaGES-9
21BR
blaOXA-56
205BR
blaSPM-1
blaOXA-56
172BR
blaSPM-1
blaOXA-56
175BR
blaSPM-1
blaOXA-56
36BR
blaOXA-56
211BR
blaOXA-56
ST277
1279293
PAO1
F1787
blaOXA-2
1260410
1280466
1265577
1271047
So098
blaOXA-2
ST244
1344658
blaCARB-2
1607533
blaOXA-2
1255885
blaKPC-2
F1757
blaVIM-2
ST654
HPA16
blaCARB-2
F2
blaVIM-2
ZG322541
blaVIM-2
Co380791
blaVIM-2
Co398373
blaVIM-2
PN586(35)w
blaVIM-2
CH3613
blaVIM-2
CH2698
blaVIM-2
Mi162
blaCARB-2
ZG8510487
blaCARB-2
blaVIM-4
ZG8525123
blaVIM-4
HPA8
blaCARB-2
ESP06B
blaCARB-2
ST111
1300097
AES-1R
LESB58
Aa249
blaOXA-129
1260990
blaVIM-2
blaOXA-10
ST395
AUS106
blaOXA-21
1275666
ESP060
blaVIM-2
ESP064
blaVIM-2
ESP084
blaVIM-2
ESP069
blaVIM-2
ESP078
blaVIM-2
ST179
1417546
1281476
1268230
1266654
1295835
1423253
4068
blaIMP-54
blaOXA-10
1301482
1271701
1252420
CH3462
blaVIM-4
PA14
ST235
1449019
1292777
1313352
1271954
CH5591
blaVEB-1
blaOXA-10
CH4877
blaVEB-1
blaOXA-10
CH2665
blaVEB-1
blaOXA-10
1324459
blaVEB-1
blaOXA-10, blaOXA-233
F2073
blaOXA-10
ZG5021922
blaIMP-7
blaOXA-2
ST357
H638
blaVEB-2
blaOXA-10
T2436 '2'
blaVEB-2
T2436
blaVEB-2, blaCARB-2
blaOXA-10
ST3986
CH4634
blaVIM-2
1254434
ST466
F1754
blaLCR-1
F2045
blaLCR-1
ZG8006959
blaVIM-2
ZG5048010
blaVIM-2
ST308
1690076
blaGES-19, blaGES-20
blaOXA-2
1420275
blaGES-19, blaGES-20
blaOXA-2
ST309
CH5174
blaCARB-2
blaVIM-1
CH4560
blaCARB-2
blaVIM-1
1275655
blaGES-19 x2, blaGES-20
blaOXA-2
PSAE2139
blaPER-1
blaOXA-2, blaOXA-74
CH2675
blaGES-5
CH2543
blaGES-5
CH4745
blaGES-1
CH5022
blaGES-5
CH2591
blaGES-1
CH5695
blaGES-1
CH4916
blaGES-5
CH2639
blaGES-1
CH2657
blaGES-5
CH5464
blaGES-5
CH5666
blaGES-1
1459372
1257147
blaOXA-1, blaOXA-17, blaOXA-129
ESP068
blaVIM-47 x2
ESP091
blaVIM-47 x2
1586994
1586981
CH5688
blaOXA-2
CH2748
blaOXA-2
MHH17441
blaOXA-2
ESP053
blaOXA-2
PSAE2127
blaOXA-2
CH2674
blaOXA-36
Vb20477
blaOXA-2
M70635118
blaVIM-1
M70638412
blaIMP-31
blaOXA-35
1260715
blaIMP-31
blaOXA-35
F2208
blaOXA-10
CH2608
blaSHV-12
CH3290
blaSHV-12
blaOXA-10
F2056
blaVIM-1
F2040
blaVIM-1
F1724
blaVIM-1
F1741
blaVIM-1
F1795
blaVIM-1
F1810
blaVIM-1
F2000
blaVIM-1
F2167
blaVIM-1
F1697
blaVIM-1
F1746
blaVIM-1
F1747
blaVIM-1
F2097
blaVIM-1
F1983
blaVIM-1
F1979
blaVIM-1
F2064
blaVIM-1
ST235

Figure S1. Phylogenetic tree of all ß-lactamase containing *P. aeruginosa* isolates.

The tree comprises 110 isolates with acquired ß-lactamases and 27 isolates with no acquired ß-lactamases (Table S3 and all ß-lactamase containing isolates from Table S2). The identities of the ß-lactamases are shown. Meropenem-resistant isolates are shown in red, meropenem-sensitive isolates in green, and isolates with unknown phenotype in black. Five *P. aeruginosa* reference strains (in blue) are also included. Sequence types (ST) are shown.

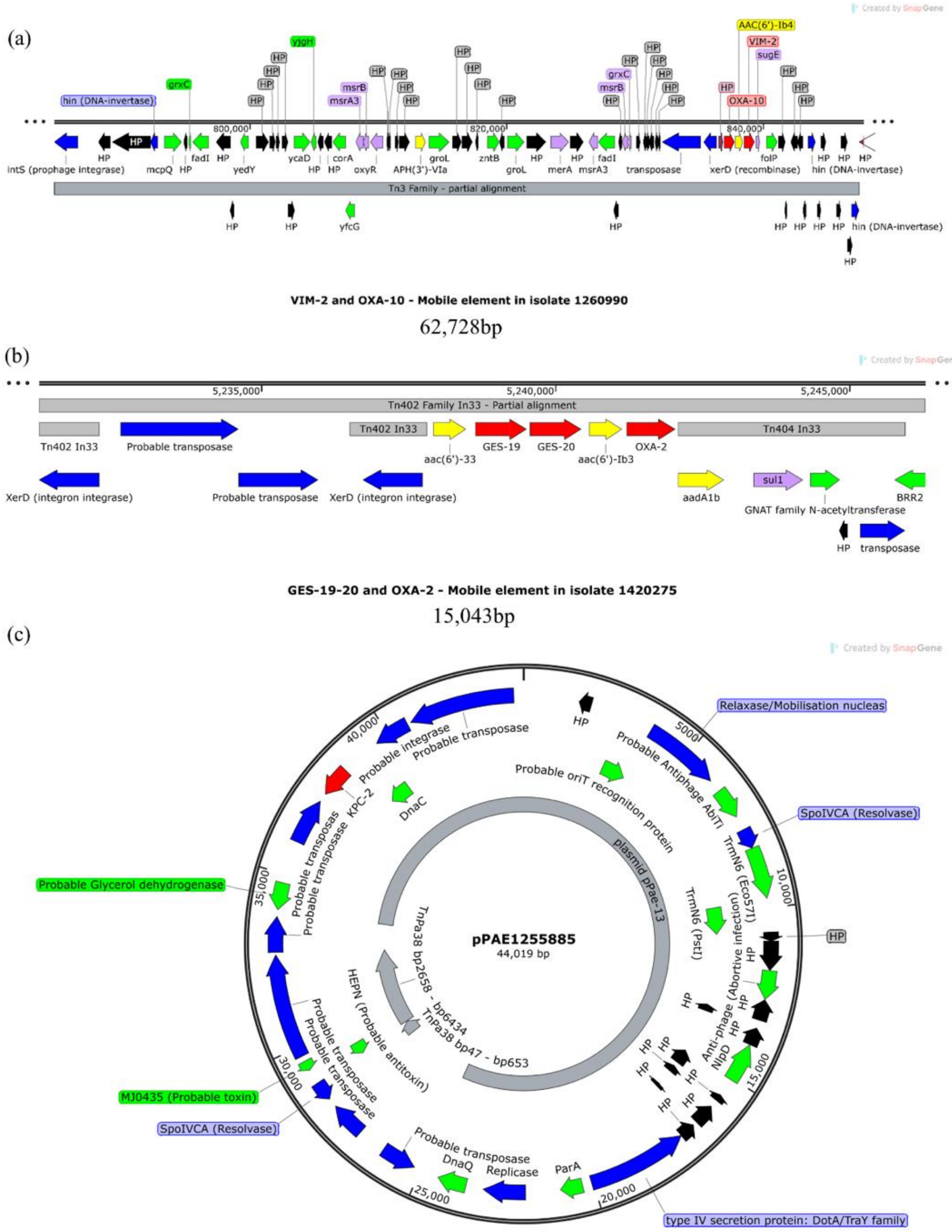

Figure S2. Maps of mobile genetic elements harbouring β-lactamases.

(a) The clinical isolate 1260990 has an uncharacterised Tn3 family mobile element harbouring the β-lactamase VIM-2 and OXA-10. (b) The clinical isolate 1420275 has an uncharacterised Tn402 family transposon that partially aligns with Tn402 In33 harbouring three β-lactamases GES-19, GES-20 and OXA-2. (c) The circular plasmid map of pPAE1255885 harbouring KPC-2.
Genes encoding β-lactamases (red), aminoglycosides (yellow), DNA mobility genes (blue), location of the mobile element (grey), other resistance genes (purple), genes with predicted functions (green) and hypothetical proteins (black) are shown. The mobile element maps were generated using SnapGene®.
